# Evaluating the impact of immune checkpoint inhibitors on liver steatosis and function: a retrospective cohort analysis

**DOI:** 10.3389/fonc.2025.1683982

**Published:** 2025-10-02

**Authors:** Sophie K. Schellhammer, Jessica Quintos Day, Run Fan, Fei Ye, Virginia Planz, Douglas B. Johnson

**Affiliations:** ^1^ Vanderbilt University School of Medicine, Nashville, TN, United States; ^2^ Department of Biostatistics, Vanderbilt University Medical Center, Nashville, TN, United States; ^3^ Department of Radiology, Vanderbilt University Medical Center, Nashville, TN, United States; ^4^ Department of Medicine, Vanderbilt University Medical Center, Nashville, TN, United States

**Keywords:** immune checkpoint inhibitor therapy, immunotherapy, liver steatosis, CT liver attenuation, immune related adverse effects (irAEs)

## Abstract

**Background:**

Immune checkpoint inhibitors (ICIs) are an essential class of immunotherapy drugs for cancer, but their impact on chronic inflammatory conditions remains unclear. Liver attenuation, a non-invasive measure of liver steatosis on CT, offers a way to assess liver inflammation. This study evaluates the impact of ICI therapy on liver attenuation and liver enzyme levels in cancer patients, building on prior research with a larger cohort.

**Methods:**

We conducted a retrospective cohort study of 164 cancer patients treated with ICIs at Vanderbilt University Medical Center between 2017 and 2022. Liver attenuation and enzyme levels (total bilirubin, AST, ALT, and alkaline phosphatase) were analyzed before and after ≥1 year of ICI therapy. Clinical factors such as weight change, liver metastasis, and steroid use were also assessed. Hepatic adverse events were characterized using CTCAE v5.0 criteria.

**Results:**

No significant changes in liver attenuation were observed from baseline to post-treatment (59.86 ± 8.07 HU vs 59.38 ± 8.36 HU, p = 0.42). Liver enzyme levels also remained stable. Post-treatment liver abnormalities occurred in 23 patients (14.0%), with most being Grade 1 elevations (11.0%). Increased body weight was significantly associated with lower liver attenuation (p < 0.0001), and liver metastasis correlated with higher total bilirubin (p < 0.001) and AST levels (p < 0.01).

**Conclusion:**

ICIs did not significantly change liver attenuation or enzyme levels, suggesting they may not exacerbate liver fat deposition or subclinical injury. Further research with additional imaging modalities is warranted.

## Introduction

Immune checkpoints serve as gatekeepers to prevent an excessive immune response that could harm host cells (1). Immune checkpoint inhibitors (ICIs) disrupt this regulatory mechanism when it has been co-opted by tumor cells and thus facilitate anti-tumor immune cell responses (2). The immune activation caused by ICIs can lead to immune-related adverse events (irAEs). Notably, immune-mediated hepatitis occurs in up to 10% of treated patients (3, 4). The exact mechanism underlying this phenomenon is unknown, but it is thought to be similar to the autoimmune-like self-reactivity found in other irAEs (5). Given the self-reactivity that can lead to irAEs in the acute setting, studies have suggested that ICI therapy could exacerbate other chronic inflammatory conditions like atherosclerosis (6–8). Mouse models and possibly preliminary human data, on the other hand, have demonstrated some anti-inflammatory benefits, including in the liver (9).

The impact of ICIs on inflammatory conditions such as metabolic dysfunction-associated steatotic liver disease (MASLD) is not well-defined (10). Investigating the impact of ICIs on liver steatosis provides an avenue to explore how ICIs impact chronic inflammatory conditions. Liver attenuation, as measured on a non-contrasted computed tomography (CT) scan, is a non-invasive measure of liver steatosis. This study builds off a previous pilot study which found trends towards improved liver attenuation and liver enzymes after ICI therapy in a much larger cohort including diverse tumor types and treatment regimens (11). Herein, we assessed a larger and more diverse cohort of long-term surviving patients and quantified changes in liver enzymes (total bilirubin, AST, ALT, and alkaline phosphatase) and liver attenuation before and after ICI initiation. To build on the prior study, we evaluated further covariates including presence of liver metastasis, baseline alcohol use, and prior treatments and compared monotherapy to combination therapy.

## Methods

This retrospective cohort analysis studied patients diagnosed with cancer and treated with anti-PD-1/PD-L1 agents at Vanderbilt University Medical Center between 2017 and 2022. Inclusion criteria required a minimum of 3 months of treatment and survival for at least 1 year from treatment initiation. Patients received anti-PD-1/PD-L1 directed agents either as monotherapy (n=118), in combination with anti-CTLA-4 (n=21), in combination with chemotherapy (n=23), or in combination with vascular endothelial growth factor inhibitors (n=2). All patients had a non-contrasted CT scan performed in isolation or in association with a PET CT scan at baseline (within 3 months of ICI initiation) and at least one year following treatment initiation. Our primary outcome was change in CT liver attenuation from baseline to ≥ one year since treatment initiation to assess whether ICI therapy was associated with a change in hepatic steatosis in cancer patients. Based on prior smaller studies suggesting hepatic stability, we hypothesized that ICIs would not significantly alter liver attenuation. Secondary outcomes included changes in liver enzymes (ALT, AST, and alkaline phosphatase) and total bilirubin, as well as characterization of post-treatment liver enzyme abnormalities according to CTCAE v5.0 grading.

Data recorded for each patient included cancer type, treatment type (agent and metastatic vs. adjuvant treatment), prior chemotherapy, presence of liver metastases, treatment duration, liver attenuation, weight, interim treatments, cancer progression, best treatment response, immunotherapy-related toxicity, use of steroids to manage toxicity, and liver enzyme levels at baseline and after at least one year of treatment. Liver attenuation was measured using Hounsfield units (HU) in SECTRA IDS 7 software. The methodology followed the Framingham Heart Study approach, a validated method of assessing liver attenuation: three 300 mm^2^ regions of interest were placed in the right lobe, posterior left lobe, and anterior left lobe at the level of the left hepatic vein entrance (12). It is important to note that the presence of liver metastasis can affect liver attenuation. To account for this, we placed our regions of interest on each scan to avoid any visualized irregularities within the liver parenchyma, such as visible metastases, which minimized the impact of any present lesions when reporting the overall liver attenuation. In addition, the presence of liver metastases was included in our chart review and was controlled for in our analysis. The final recorded liver attenuation value was an average of the three regions.

Continuous variables were compared using the Wilcoxon signed rank test for paired data. Linear mixed-effects models were used to evaluate changes in liver attenuation and enzymes. Models included age, metastatic status, presence of liver metastases, prior therapy, ICI class, steroid requirement for toxicity, weight, time, and a weight-by-time interaction term as fixed effects, with subject specified as a random effect to account for repeated measures at scans 1 and 2. Insignificant interaction terms (p > 0.05) between weight and time were removed from the final model. Skewed outcomes (total bilirubin, AST, ALT, and alkaline phosphatase) were log10-transformed prior to fitting the regression models.

Assumptions for linear mixed-effects models were evaluated to ensure model validity. Quantile-quantile plots were used to check normal distribution of residuals and random intercepts, and residual plots were used to further confirm homoscedasticity. The residuals and random intercepts were approximately normally distributed, and the variances remained constant across fitted values without evident patterns. Together, these assumption checks support the appropriateness of the linear mixed-effects modeling for the data.

Among all patients (n = 164), two covariates—metastatic status and liver metastases—had missing values: metastatic status was missing in two patients and liver metastases in one patient, accounting for less than 2% of the cohort. All other covariates were complete. The missing data were minimal and unlikely to introduce bias or affect the robustness of the results. Given this low level of missingness, we did not apply multiple imputations and instead performed complete case analysis.

## Results

**Figure 1:**
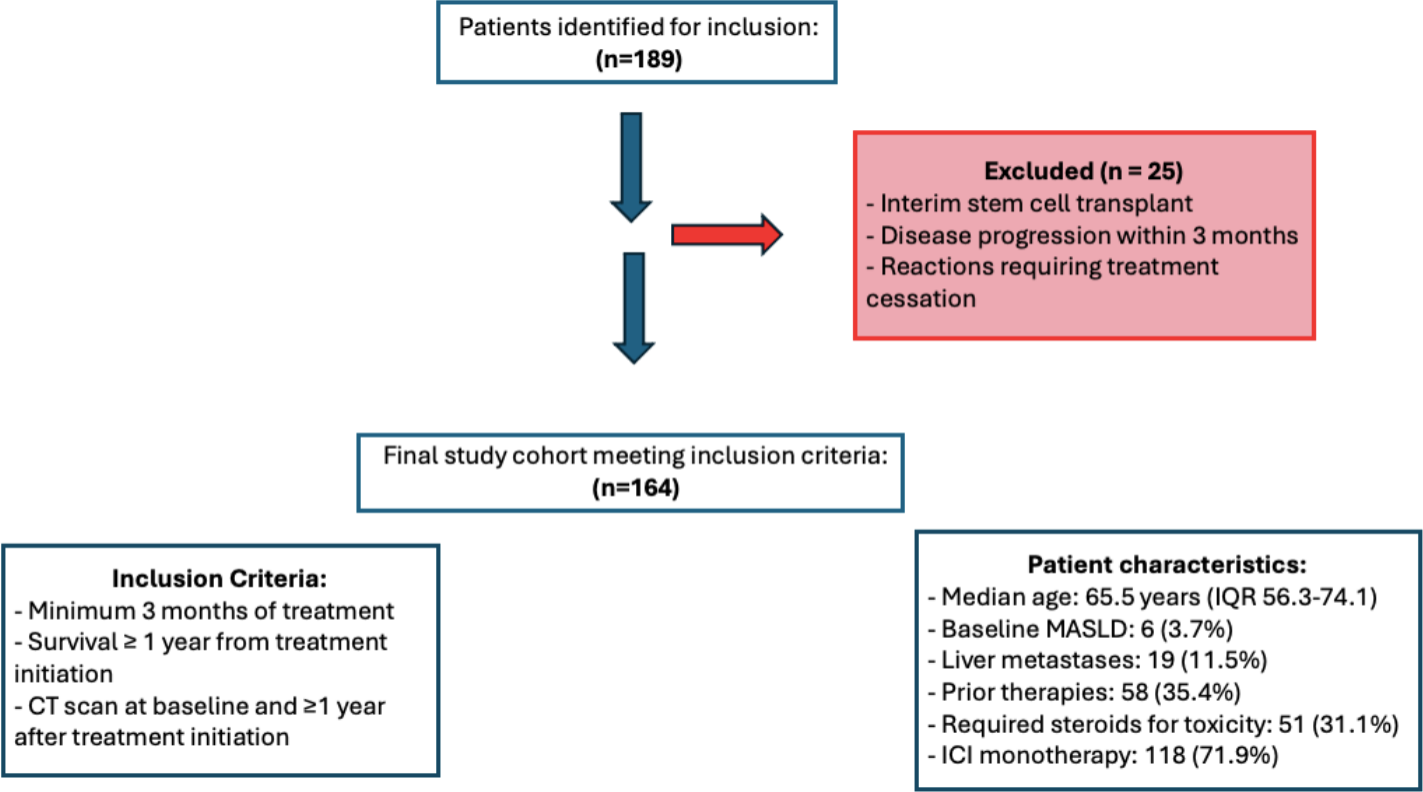
Cohort flow diagram.

A total of 189 patients were identified; 25 patients were excluded because of interim stem cell transplant, disease progression within 3 months, or reactions requiring treatment cessation (**Figure 1**). The remaining 164 patients had a median age of 65.5 years (interquartile range [IQR] of 56.3 to 74.1 years). Of the patients, 58 (35.3%) were female and there was an equal number of patients with adjuvant and metastatic conditions treatment (n=82 for both). Nineteen patients (11.5%) had liver metastases and 35.4% (n=58) received prior therapies; 31.1% (n=51) required steroids for toxicity. Among the cancer types, melanoma was the most common (32.1%, n=53), followed by non-small cell lung cancer (NSCLC) (26.1% n=43) and renal cell carcinoma (11.5%, n=19). Most patients received immune checkpoint inhibitor (ICI) monotherapy, comprising 71.9% (n=118). Combination therapy was administered to 12.8% (n=21) of patients, 14.0% (n=23) received ICI/chemotherapy, and 1.2% (n=2) were treated with ICI/VEGF inhibitors. Median baseline weight measurements were 83.2kg (IQR 69.6 - 102.8kg). Metabolic dysfunction-associated steatotic liver disease (MASLD) was diagnosed in 3.7% (n=6) of patients (**Table 1**). During the study period, 6.7% (n=11) of patients developed ICI-hepatitis. These patients were retained in the analysis as they represent clinically relevant liver changes associated with ICI therapy.

**Table 1:** Patient characteristics.

Characteristics	Category	Value
Age, *years*	Median (IQR)	65.5 years (56.3-74.1 years)
Gender, *n*	Male	106
	Female	58
Adjuvant vs Metastatic, *n*	Adjuvant	82
	Metastatic	82
Cancer type, % (n)*	Melanoma	32.1% (53)
	Non-small cell lung cancer	26.1% (43)
	Other*	11.5% (19)
	Renal cell carcinoma	11.5% (19)
	Urothelial carcinoma	6.1% (10)
	Head and neck cancers	5.5% (9)
	Esophageal cancers	3.6% (6)
	Hodgkin lymphoma	3.6% (6)
Therapy, *n*	ICI monotherapy	118
	ICI Combination therapy	21
	ICI/Chemo	23
	ICI/VEGF	2
Baseline weight, *kg*	Median (IQR)	83.2 kg (69.6 kg-102.8 kg)
Diagnosed MASLD, n	Yes	6
	No	158

*one patient had both urothelial carcinoma and renal cell carcinoma.

**Includes cancer types with less than five cases: Merkel cell carcinoma (4), small cell lung cancer (3), hepatocellular carcinoma (2), cutaneous squamous cell carcinoma (4), breast cancer (1), carcinoma of unknown primary (1), cholangiocarcinoma (1), colorectal carcinoma (1), endometrial adenocarcinoma (1), large cell rectal neuroendocrine carcinoma (1).

**Figure 2:**
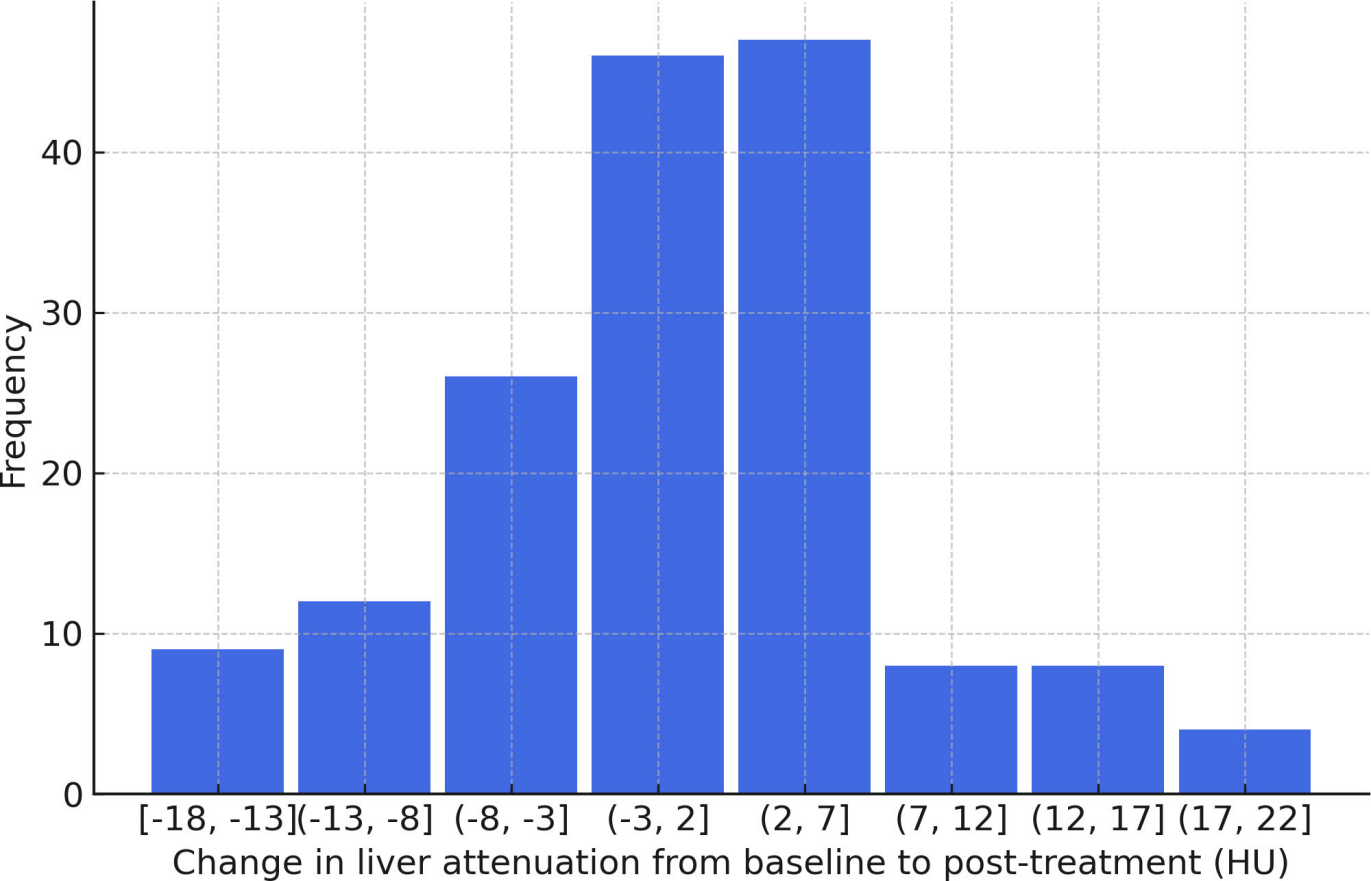
Distribution of change in liver attenuation following immunotherapy treatment.

There was no significant difference in liver attenuation from baseline to at least one year after treatment, with mean values of 59.86 ± 8.07 HU pre-treatment and 59.38 ± 8.36 HU post-treatment (p = 0.42) (**Figure 2**). Similarly, no significant changes were observed in liver function parameters. Total bilirubin levels showed a slight increase from 0.50 ± 0.35 to 0.61 ± 0.57, though this change did not reach statistical significance (p = 0.065). Aspartate aminotransferase (AST) levels were 25.2 ± 18.4 pre-treatment and 23.6 ± 15.1 post-treatment (p = 0.82). Alanine aminotransferase (ALT) remained relatively stable as well, with values of 24.1 ± 24.8 pre-treatment and 23.9 ± 22.5 post-treatment (p = 0.86). Alkaline phosphatase levels also showed minimal variation, from 92.4 ± 38.7 pre-treatment to 95.3 ± 53.7 post-treatment (p = 0.74) (**Table 2**). An exploratory univariate analysis compared changes in liver attenuation between patients receiving monotherapy versus combination therapy. Patients receiving monotherapy (n = 118) had a mean ΔHU of 0.44 ± 9.86 (median 0.00), while those receiving combination therapy (n = 46) had a mean ΔHU of -1.38 ± 6.06 (median -2.14). A two-tailed Mann–Whitney U test did not indicate a statistically significant difference between groups (z = 1.26, p = 0.208). Additional analyses were completed to evaluate the six patients with diagnosed MASLD, with mean liver attenuation on the pre-treatment scan of 50.8 ± 12 HU and 51.0 ± 8.48 HU at the post-treatment scan. As for liver enzymes, this group had a bilirubin of 1.33 ± 1.19 pre-treatment and 1.02 ± 1.13 post-treatment, AST of 52.5 ± 39.2 pre-treatment and 22.8 ± 8.35 post-treatment, and ALT of 90 ± 91.7 pre-treatment and 30.7 ± 24.3 post-treatment.

**Table 2:** Liver attenuation and liver enzymes before and after treatment.

Variable	Pre-treatment	Median (IQR)	Post-treatment	Median (IQR)	P-value
Mean liver attenuation (HU)	59.86 ± 8.07	61.58 (56.64-65.60)	59.38 ± 8.36	59.71 (55.13-65.64)	0.42
Mean total bilirubin	0.50 ± 0.35	0.40 (0.30-0.60)	0.61 ± 0.57	0.50 (0.30-0.70)	0.065
Mean AST	25.2 ± 18.4	20.0 (15.0-26.0)	23.6 ± 15.1	20.0 (15.0-28.0)	0.82
Mean ALT	24.1 ± 24.8	19.0 (12.8-26.0)	23.9 ± 22.5	18.0 (13.0-26.0)	0.86
Mean Alkaline Phosphatase	92.4 ± 38.7	84 (68.0-103.0)	95.3 ± 53.7	80.5 (69.0-104.2)	0.74

We then assessed what clinical features correlated with liver attenuation and enzyme changes between baseline and at least one year follow up. Increased weight was significantly associated with lower liver attenuation (p < 0.0001). Higher total bilirubin levels were observed in patients with liver metastasis (p < 0.001), those who did not require steroids (p = 0.037), and those with higher post-treatment weight (p = 0.050); prior therapies were associated with lower total bilirubin levels (p = 0.014). Metastatic therapy was associated with lower AST levels (p = 0.049), while liver metastasis corresponded with higher AST (p < 0.01). ALT levels were negatively correlated with age (p < 0.001). Alkaline phosphatase levels were higher in patients with liver metastasis (p < 0.001) but lower in patients with higher weight (p = 0.049) (**Table 3**).

**Table 3:** Linear mixed-effect model for liver attenuation and liver enzyme outcomes (n=164).

Variable	Liver attenuation		Total bilirubin		AST		ALT		Alkaline phosphatase	
	Coefficient [95% CI]	P-value	Coefficient [95% CI]	P-value	Coefficient [95% CI]	P-value	Coefficient [95% CI]	P-value	Coefficient [95% CI]	P-value
Age	0.018 [-0.061, 0.097]	0.66	0.002 [-0.0003, 0.004]	0.087	-0.0011 [-0.003, 0.0009]	0.30	-0.0051 [-0.0076, -0.0026]	0.0001*	-0.0002 [-0.0018, 0.0014]	0.84
Metastatic cancer (Metastatic: Adjuvant)	-0.84 [-3.12, 1.44]	0.48	-0.066 [-0.13, 0.00002]	0.054	-0.060 [-0.12, -0.0013]	0.049*	-0.041 [-0.11, 0.03]	0.26	0.0079 [-0.038, 0.054]	0.74
Liver metastases (Yes: No)	-1.48 [-4.89, 1.93]	0.40	0.18 [0.083, 0.28]	0.0005*	0.12 [0.031, 0.21]	0.0096*	0.084 [-0.023, 0.19]	0.13	0.12 [0.053, 0.19]	0.0008*
Use of prior therapies (Yes: No)	0.83 [-1.53, 3.19]	0.50	-0.087 [-0.15, -0.019]	0.0140*	0.028 [-0.032, 0.089]	0.37	-0.0079 [-0.081, 0.065]	0.83	0.037 [-0.011, 0.084]	0.13
Class										
Ipilimumab or nivolumab: PD1 monotherapy	0.43 [-3.14, 4.00]	0.82	0.011 [-0.093, 0.11]	0.84	-0.033 [-0.13, 0.060]	0.49	-0.0055 [-0.12, 0.11]	0.92	0.042 [-0.03, 0.11]	0.26
ICI + chemotherapy or VEGF: PD1 monotherapy	0.83 [-2.26, 3.92]	0.60	0.059 [-0.031, 0.15]	0.20	0.033 [-0.048, 0.11]	0.43	0.041 [-0.057, 0.14]	0.42	0.045 [-0.018, 0.11]	0.17
Steroids for toxicity (None: Any)	-0.42 [-2.84, 1.99]	0.73	0.076 [0.006, 0.15]	0.037*	0.030 [-0.032, 0.092]	0.35	0.0068 [-0.068, 0.082]	0.86	0.037 [-0.011, 0.086]	0.14
Weight at procedure	-0.12 [-0.16, -0.071]	<0.0001*	0.0003 [-0.001, 0.002]	0.70	-0.0008 [-0.0019, 0.0003]	0.18	0.00004 [-0.0013, 0.0014]	0.95	-0.0009 [-0.0018, -0.000017]	0.049*
Visit (Visit 2: Visit 1)	-0.61 [-1.82, 0.60]	0.33	-0.079 [-0.21, 0.053]	0.25	-0.021 [-0.054, 0.013]	0.23	-0.0067 [-0.051, 0.037]	0.77	-0.0022 [-0.027, 0.023]	0.87
Weight at procedure * Visit	—–	—–	0.0015 [0.00003, 0.003]	0.0498*	—–	—–	—–	—–	—–	—–

*p < 0.05.

Post-treatment liver abnormalities according to CTCAE v5.0 criteria were observed in 23 patients (14.0%) in our cohort. Most of these abnormalities were mild, with 18 patients (11.0%) experiencing Grade 1 elevations. Grade 2 abnormalities occurred in three patients (1.8%), consisting of two patients with isolated bilirubin elevation and one patient with isolated ALT elevation. Two patients (1.2%) developed Grade 2–3 mixed pattern abnormalities. Of the three patients who had greater than Grade 1 injury that was not an isolated bilirubin elevation, one had a grade 2 elevation in alkaline phosphatase and grade 3 elevation in total bilirubin, one had grade 3 AST, ALT, and total bilirubin elevation and a grade 2 alkaline phosphatase elevation, and the last patient had a grade 2 elevation of ALT. Calculation of the R factor for these three patients revealed that two had a hepatocellular pattern of liver injury and one had a cholestatic patter of liver injury (**Table 4**).

**Table 4:** Post-treatment liver abnormalities according to CTCAE v5.0 guidelines.

CTCAE v5.0 grade	%* (n)	Liver injury pattern by R factor**
Grade 1	11.0% (18)	10 cholestatic, 2 mixed
Grade 2	1.8% (3)	1 hepatocellular
Grade 2 and 3 mixed injury	1.2% (2)	1 cholestatic, 1 hepatocellular

*Percentages based on the total cohort of 164 patients.

**R factor not calculated for patients with isolated bilirubin or AST elevations.

•R factor = (ALT/ULN) ÷ (ALP/ULN); ≥5 = hepatocellular, ≤2 = cholestatic, 2-5 = mixed; using institutional ULN cutoff.

**Figure 3:**
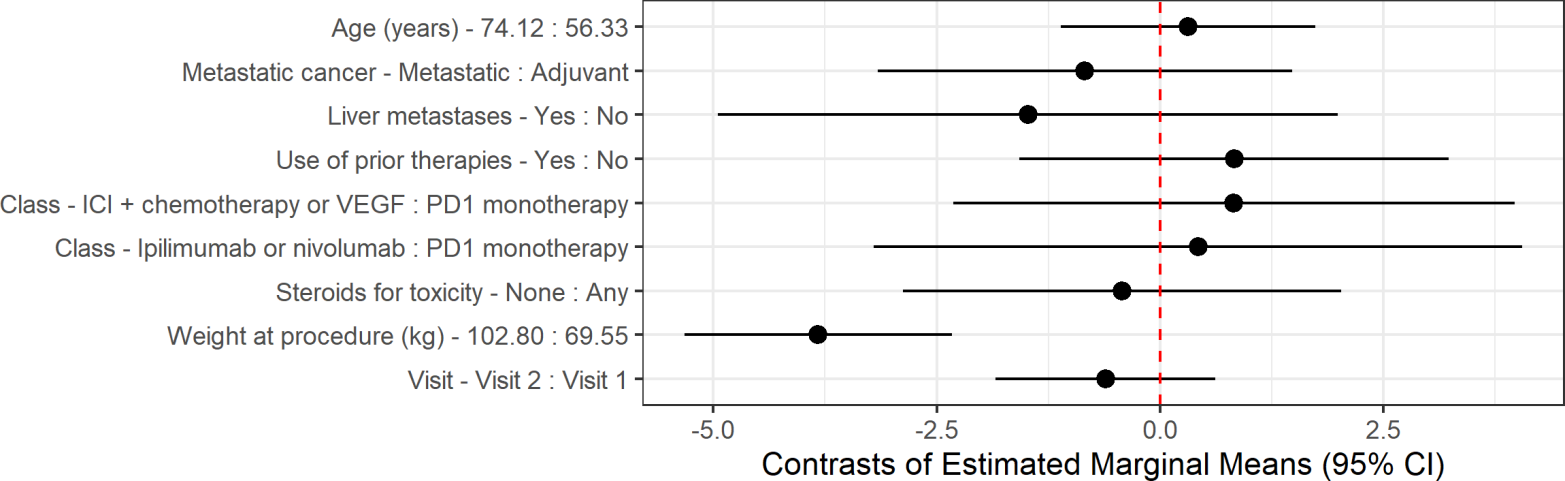
Contrast of estimated marginal means (EMMs) for liver attenuation outcome. Estimates and 95% confidence intervals (CIs) are derived from linear mixed-effects models adjusted for all covariates shown, with subject as a random effect. For continuous variables, contrasts are calculated between the 75^th^ and 25^th^ percentiles. For categorical variables, contrasts are calculated between groups. Points represent EMMs differences, and horizontal lines indicate 95% CI. The dashed vertical line at 0 indicates no difference.

An updated linear mixed-effects model was created to incorporate baseline alcohol consumption, with no significant effect on bilirubin (p=0.16), AST (p=0.17), or ALT (p=0.17) noted (**Figure 3**). Patients who received other treatments during the study period were also included in the updated linear mixed-effects model, with no significant effect on liver attenuation (p=0.061), bilirubin (p=0.80), AST (p=0.57), or ALT (p=0.48). As the inclusion of baseline alcohol consumption and additional treatments did not significantly alter the primary outcomes, we have retained the original linear mixed-effects model as the primary analysis for consistency with the study’s original framework.

Lastly, a sub analysis of patients with liver enzymes elevated at baseline was performed. Due to the small sample size (n=25), only visit was included as fixed effect in univariable linear mixed-effects model. AST (p=0.0011) and alkaline phosphatase (p=0.0219) showed a significant decrease over time, while ALT exhibited a marginally significant decrease (p=0.0543).

## Discussion

The results of this study suggest that ICIs do not significantly impact liver attenuation or liver enzyme levels and thus do not demonstrate an obvious impact of ICI on fat deposition or liver injury. While earlier pilot work in melanoma suggested stability of liver attenuation and enzymes with ICIs, our study confirms and extends these findings in a larger, more diverse cohort spanning multiple cancer types. By incorporating metastatic versus adjuvant therapy type, presence of liver metastases, prior systemic therapies, baseline alcohol use, and combination regimens beyond PD-1 monotherapy, we provide a more comprehensive evaluation of hepatic effects. This broader scope enhances generalizability and strengthens the evidence that ICIs are not associated with clinically meaningful hepatic changes across varied patient populations. We found that increased weight was significantly associated with lower liver attenuation, however even these patients did not have obvious worsening of liver attenuation while on ICI treatment (13). Interestingly, however, patients with pre-existing MASLD or elevated AST/ALT did have improvement in their liver enzymes over the course of the study, a finding which should be validated as the sample size analyzed here was very small and thus susceptible to random fluctuations. This intriguing observation could suggest that immune checkpoint inhibitor therapy reduces hepatic stress through modulation of inflammatory pathways or improved metabolic profiles secondary to cancer treatment. However, alternative explanations such as resolution of pre-treatment liver injury, regression to the mean in patients with baseline elevations, or changes in concomitant medications cannot be excluded.

Clinical factors such as liver metastases, steroid use, prior therapy, baseline and post-treatment weight, and age were associated with significant variations in liver enzymes. Patients with liver metastases were found to have increased total bilirubin, AST, and alkaline phosphatase levels, a trend identified in prior studies and likely explained by the structural disruption caused by liver metastases (14–16). Our study found that patients who did not receive steroids to manage ICI-related toxicity had higher total bilirubin levels. Steroids are known to decrease serum total bilirubin concentrations and treat liver inflammation and may have lowered the total bilirubin levels of the steroid recipients (17). Interestingly, patients who received prior therapies were found to have lower total bilirubin levels. Prior therapies may have “conditioned” the liver, enhancing its resilience to subsequent treatments and resulting in lower total bilirubin levels in patients who have undergone previous treatments. Finally, given that patients in this study received a variety of treatment regimens during the study period, an exploratory univariate analysis comparing patients who received ICI monotherapy with patients who received combination therapy was performed and showed no significant difference in liver attenuation between the two groups.

The study also examined the liver enzymes AST, ALT, and alkaline phosphatase. Increasing age was found to be significantly associated with decreased ALT levels. Low ALT is a known indicator of sarcopenia and frailty (18, 19). This observation aligns with previous studies suggesting that ALT levels decrease after age 65 (20, 21). Our study also demonstrated that increased weight was associated with lower alkaline phosphatase levels. This is unexpected, as obesity has been shown to correlate with increasing alkaline phosphatase levels in mice and people (22, 23). This unexpected inverse relationship may reflect that cancer patients with lower weight are more likely to have advanced disease, malnutrition, or metastatic involvement of the liver or bone, all of which are associated with elevated alkaline phosphatase levels, whereas patients with higher weight may have less severe disease and correspondingly lower alkaline phosphatase. Elevated alkaline phosphatase is a recognized marker of cancer burden and poor prognosis, particularly in the context of involuntary weight loss and metastatic disease (24, 25). At the same time, this could be a spurious finding given that alkaline phosphatase is known to fluctuate across the lifespan and should be measured fasting for the most accurate results (26, 27). Overall, the associations identified between liver enzymes and clinical factors appear to reflect underlying patient factors and expected physiological and pathological relationships rather than ICI-related impacts. This may inform clinical decision-making by identifying patient subgroups that warrant closer hepatic monitoring during ICI therapy, particularly those with liver metastases.

It is important to acknowledge the limitations of our study. First, the heterogeneity of ICI regimens and cancers in our cohort limited our ability to detect regimen or cancer type-specific hepatic effects due to insufficient sample sizes within individual categories. Second, our imaging approach had inherent limitations. For pragmatic reasons, we used CT scan measurements (all patients were receiving these for their cancer treatment) rather than biopsies or other modalities like MRI. CT attenuation is subject to inter-reader differences and machine variability but, unfortunately, more sensitive modalities such as Magnetic Resonance Imaging Proton Density Fat Fraction or elastography were rarely performed, if at all, in our patient cohort. Future research should incorporate these advanced imaging modalities to provide more sensitive assessment of liver health. Third, our laboratory data collection was not standardized with respect to fasting status, though all values were collected and processed within the same laboratory system, reducing inter-lab variability. Additionally, because liver health is multifactorial, determining whether changes were specifically related to ICIs versus other factors remains challenging. We controlled for weight change, alcohol use, steroid use, liver metastasis, and prior therapies, but we lacked systematic data on viral hepatitis status, lipid profiles, and concomitant hepatotoxic medications and did not collect data on diabetes or other metabolic syndromes, which represent unmeasured confounders. Finally, our design introduced survivorship bias by requiring patients to survive at least one year, likely underestimating early ICI-related hepatotoxicity and limiting the generalizability of our findings to patients who tolerate initial therapy. Future studies should incorporate patients who terminated treatment early and also include extended follow-up periods with a greater number of patients having pre-existing liver conditions.

## Conclusion

Our study provides evidence that ICI therapy does not have a substantial impact on liver attenuation or enzymes in ICI therapy recipients. While factors like weight, liver metastasis, and age were associated with liver enzyme variations, these findings do not suggest an overarching effect of ICIs on liver fat deposition or subclinical liver injury.

## Data Availability

The original contributions presented in the study are included in the article/supplementary material. Further inquiries can be directed to the corresponding author.
